# MicroRNA miR-301a is a novel cardiac regulator of Cofilin-2

**DOI:** 10.1371/journal.pone.0183901

**Published:** 2017-09-08

**Authors:** Ashraf Yusuf Rangrez, Phillip Hoppe, Christian Kuhn, Elisa Zille, Johanne Frank, Norbert Frey, Derk Frank

**Affiliations:** 1 Department of Internal Medicine III (Cardiology, Angiology, Intensive Care), University Medical Center Kiel, Kiel, Germany; 2 DZHK (German Centre for Cardiovascular Research), partner site Hamburg/Kiel/Lübeck, Kiel, Germany; Texas A&M University Health Sciences Center, UNITED STATES

## Abstract

Calsarcin-1 deficient mice develop dilated cardiomyopathy (DCM) phenotype in pure C57BL/6 genetic background (Cs1-ko) despite severe contractile dysfunction and robust activation of fetal gene program. Here we performed a microRNA microarray to identify the molecular causes of this cardiac phenotype that revealed the dysregulation of several microRNAs including miR-301a, which was highly downregulated in Cs1-ko mice compared to the wild-type littermates. Cofilin-2 (Cfl2) was identified as one of the potential targets of miR-301a using prediction databases, which we validated by luciferase assay and mutation of predicted binding sites. Furthermore, expression of miR-301a contrastingly regulated Cfl2 expression levels in neonatal rat ventricular cardiomyocytes (NRVCM). Along these lines, Cfl2 was significantly upregulated in Cs1-ko mice, indicating the physiological association between miR-301a and Cfl2 *in vivo*. Mechanistically, we found that Cfl2 activated serum response factor response element (SRF-RE) driven luciferase activity in neonatal rat cardiomyocytes and in C2C12 cells. Similarly, knockdown of miR301a activated, whereas, its overexpression inhibited the SRF-RE driven luciferase activity, further strengthening physiological interaction between miR-301a and Cfl2. Interestingly, the expression of SRF and its target genes was strikingly increased in Cs1-ko suggesting a possible *in vivo* correlation between expression levels of Cfl2/miR-301a and SRF activation, which needs to be independently validated. In summary, our data demonstrates that miR-301a regulates Cofilin-2 *in vitro* in NRVCM, and *in vivo* in Cs1-ko mice. Our findings provide an additional and important layer of Cfl2 regulation, which we believe has an extended role in cardiac signal transduction and dilated cardiomyopathy presumably due to the reported involvement of Cfl2 in these mechanisms.

## Introduction

Several forms of cardiomyopathies including dilated (DCM), hypertrophic (HCM), and ischemic cardiomyopathy (ICM), can lead to heart failure which is linked to poor prognosis [1, 2]. DCM patients suffer from dilatation of the left ventricle, hypertrophy of the cardiomyocytes with increased heart weight and myocardial fibrosis, arrhythmias, heart failure and a high risk for sudden cardiac death; however, the etiology of the DCM is not sufficiently understood [3, 4]. Possible causes though incorporate genetic and non-genetic variables whereby the genetic background is suspected to be responsible for up to 50% of all DCM cases. For better understanding of the pathophysiology of cardiomyopathy, it is crucial to identify and characterize the biomolecules and molecular pathways that are involved in the pathogenesis [2, 5, 6]. One of the well characterized pathways is Calcineurin signaling which has been shown to be involved in the development of pathological HCM and DCM [7–9]. Previously, the Calsarcin protein family has been described as Calcineurin-interacting proteins at the Z-disk in muscle cells [10–12]. Calsarcin-1 is the only isoform present in the adult heart [12], which tethers Calcineurin to α-actinin at the Z-disc and inhibits pathological hypertrophic response due to Calcineurin [13]. Calsarcin-1 also prevents angiotensin-II induced cardiac hypertrophy in a cardiac-specific transgenic mouse model, which underlines its role as an inhibitor of pathological hypertrophy [14]. In contrast, Calsarcin-1 deficient mice are sensitized to Calcineurin signaling and show a massive hypertrophic cardiomyopathy when stressed biomechanically in a mixed genetic background without displaying a baseline hypertrophy phenotype [13]. In present study, after more than 10 back-crosses to obtain Calsarcin-1 null mouse in pure C57BL/6 background in order to study the effect of genetic background, if any, mice displayed dilated cardiomyopathy phenotype with contractile dysfunction and increased expression of fetal genes *nppa*, and *nppb* in addition to upregulated *rcan1-4* without any signs of hypertrophy.

The tread-milling of actin and its regulation via RhoA, a small family GTPase, and transcription factor serum response factor (SRF), when dysfunctional, highly suggestive of DCM [15–18]. The treadmilling and actin dynamic is also regulated by the Actin-depolymerizing factor (ADF)/cofilin family proteins. This protein family consists of three members in mammals, Cofilin-1 which is expressed in all cell types, ADF is expressed only in epithelial/endothelial cells, and Cofilin-2 (Cfl2), which is predominantly expressed in the heart and skeletal muscle [19–23]. The structure of ADF/cofilin family protein is evolutionarily highly conserved suggesting an essential structural and functional role for these proteins [24]. One of its main functions is the regulation of actin dynamics by binding and severing filamentous actin at the pointed end (-) [25]. Especially, Cfl2, the muscle isoform, reportedly control precise length of the sarcomeres in cardiomyocytes [26]. Lack of Cfl2 in mice is not embryonically lethal indicating no significant developmental role, however, Cfl2 deficient mice die around 8 days after birth due to severe muscle defects [27]. Interestingly, Cfl2 has recently been linked to the pathogenesis of DCM where Cfl2 was found to be hyper-phosphorylated and present in aggregates in cardiomyocytes of human idiopathic DCM samples [28]. Furthermore, the heterogenic knockout of Cfl2 in the murine heart leads to a DCM-like phenotype [28]. Nevertheless, Cfl2 function and its regulation are complex [21] and its precise role in the development of DCM is not satisfactorily understood.

Here, we performed a microRNA microarray on cardiac RNA extracted from Calsarcin-1 deficient mice in a pure C57BL/6 background, which display a strict DCM phenotype. Through this screen, we identified miR-301a as the most downregulated microRNA that we found targets Cfl2, a major regulator of actin dynamics, *in vitro* and *in vivo*. Moreover, our *in vitro* data indicated that miR-301a attenuates RhoA-mediated activation of SRF signaling via targeting Cfl2 without affecting cellular hypertrophy. Therefore, we believe that our findings provide an important layer in Cfl2 regulation, which needs further *in vivo* validations for exploiting the therapeutic potential of miR-301/Cfl2 interactions in cardiac signal transduction and DCM.

## Materials and methods

### Generation and characterization of Calsarcin-1 knock out in C57BL/6 pure background

The Calsarcin-1 knockout mice were originally created and characterized in 2004 by Frey et al. in a mixed background [13]. The backcrossing in a pure C57BL/6NCr background was performed by Schoensiegel et al. in 2007 [29]. Primer pairs used for the genotyping were: neo_F: 5’-gat gcg gtg ggc tct atg gct tct gag gc-3’, CS1_F: 5’-cag tgt gtt cta tta ccc agg ctg tc-3’ and CS1_R: 5’-gtc ctc aca act aat tca tgt aca gat g-3’. All the animal experiments were carried out in strict accordance to the ethical guidelines by MELUR (Ministry of Energy, Agriculture, the Environment and Rural Areas). Mice were given access to the food and water ‘*ad libitum*’ and maintained under 12 h dark and light cycle temperature and air controlled rooms.

### Echocardiography

Echocardiography was carried out on anaesthetized mice with Isoflurane (2.5 ppm) on Vivid 7 Pro Ultrasound System (GE healthcare). The examiner was blinded for the genotype. Mice were killed immediately after the echocardiography by cervical dislocation and organs were harvested, weighed and stored at -80°C until further processing.

### RNA isolation, cDNA synthesis and real-time PCR

Heart tissue samples were shredded using the tissue separator (IKA Ultra-Turrax; Sigma-Aldrich) in 1ml QIAzol (Qiagen). Total RNA from neonatal rat ventricular cardiomyocytes (NRVCM) was also isolated using QIAzol (Qiagen) according to manufacturer’s instructions. Contaminating DNA was digested using DNase I (Thermo Fisher Scientific) and total RNA concentration was measured on NanoDrop spectrophotometer (Thermo Fisher Scientific). cDNA was synthesized using 1μg total RNA with hexanucleotide random-primer-mix (Carl Roth) and SuperScript III cDNA synthesis kit (Thermo Fisher Scientific). Quantitative real-time PCR (qRT-PCR) was performed on CFX96 Real-Time PCR system (Bio-Rad Laboratories) using 10ng of cDNA with EXPRESS SYBR® GreenER™ (Thermo Fisher Scientific). PCR conditions used were, initial denaturation at 95°C for 30 seconds followed by 40 cycles of a denaturation step at 95°C for 5 seconds, and a common primer hybridization, elongation, and data collection step at 60°C for 30 seconds. Following primers were used: RPL32mr_165_R 5’-ccg cac cct gtt gtc aat gc-3’, RPL32mr_165_F 5’-ggt ggc tgc cat gtg ttt tac g-3’, Cofilin‑2_m_F 5’-ccg acc cct cct tct tct cg-3’ (mouse), Cofilin‑2_m_R 5’-gta act cca gat gcc ata gtg c-3’ (mouse), Cofilin‑2_r_F 5’-gca gat ctt ggt ggg tga ca-3’ (rat), Cofilin‑2_r_R 5’-cac ttt cag gag ccc aga ata taa-3’ (rat). The iQ™ Multiplex Powermix (Bio-Rad Laboratories) was used for the multiplex qRT-PCR to simultaneously measure *nppa*, *nppb* and *rcan1-4* expression. The primer pairs were: Nppa_F: 5’-gga gca aat cct gtg tac agt g-3’, Nppa_R: 5’-acc tca tct tct acc ggc at-3’, Nppb_F: 5’-aca aga tag acc gga tcg ga-3’, Nppb_R: 5’-agc cag gag gtc ttc cta ca-3’, Rcan1‑4_F: 5’-tag ctc cct gat tgc ttg tg-3’, Rcan1‑4_R: 5’-gga ttc aaa ttt ggc cct gg-3’, Rpl32_F: 5’-ctg ctg atg tgc aac aaa tct-3’, Rpl32_R: 5’-gct gtg ctg ctc ttt cta caa t-3’. Additional probes were added, which are labeled with specific fluorophores and quencher: Nppa_probe FAM‑5’-tga tgg att tca aga acc tgc tag acc a-3’‑BHQ1, Nppb_probe HEX‑5’-tca gtg cgt tac agc cca aac ga-3’‑BHQ1, Rcan1‑4_probe Cy5.5‑5’-acg atg atg tct tca gcg aaa gtg aga c-3’‑Eclipse, Rpl32_probe Texas Red‑5’-act gtg ctg aga ttg ctc aca atg tgt-3’‑BHQ2.

### Microarray analysis

#### RNA preparation and hybridization

Total RNA from Cs1-ko and wild-type mice was isolated using QIAzol lysis reagent according to the manufacture's instruction (Qiagen). The quality of total RNA was checked by gel analysis using the total RNA Nano chip assay on an Agilent 2100 Bioanalyzer (Agilent Technologies). Only samples with RNA index values >8.5 were selected for expression profiling. RNA concentrations were determined using the NanoDrop spectrophotometer (NanoDrop Technologies). Biotin-labeled cRNA samples for hybridization on Illumina Mouse Sentrix-6 BeadChip arrays (Illumina, Inc.) were prepared according to Illumina's recommended sample labeling procedure based on the modified Eberwine protocol [30]. In brief, 250 ng total RNA was used for complementary DNA (cDNA) synthesis, followed by an amplification/labeling step (*in vitro* transcription) to synthesize biotin-labeled cRNA according to the MessageAmp II RNA Amplification kit (Ambion, Inc.). The cRNA was column purified according to TotalPrep RNA Amplification Kit, and eluted in 60 μl of water. Quality of cRNA was controlled using the RNA Nano Chip Assay on an Agilent 2100 Bioanalyzer and spectrophotometrically quantified (NanoDrop). Hybridization was performed at 58°C, in GEX-HCB buffer (Illumina Inc.) at a concentration of 100 ng cRNA/μl, unsealed in a wet chamber for 20h. Spike-in controls for low, medium and highly abundant RNAs were added, as well as mismatch control and biotinylation control oligonucleotides. Microarrays were washed twice in E1BC buffer (Illumina Inc.) at room temperature for 5 minutes. After blocking for 5 min in 4 ml of 1% (wt/vol) Blocker Casein in phosphate buffered saline Hammarsten grade (Pierce Biotechnology), array signals were developed by a 10-min incubation in 2 ml of 1 μg/ml Cy3-streptavidin (Amersham Biosciences) solution and 1% blocking solution. After a final wash in E1BC, the arrays were dried and scanned.

#### Scanning and data analysis

Microarray scanning was carried out using a Beadstation array scanner, with settings adjusted to a scaling factor of 1 and PMT settings at 430. Data extraction was done for all beads individually, and outliers were removed when > 2.5 MAD (median absolute deviation). All remaining data points were used for the calculation of the mean average signal for a given probe, and standard deviation for each probe was calculated. Scanning data was analyzed by normalization of signals using the quantile normalization algorithm without background subtraction, and differentially regulated genes were defined by calculating the standard deviation differences of a given probe in Cs1-ko vs WT mice comparison. Microarray data is deposited to GEO databank under the accession number GSE100851.

### Protein isolation and immunoblotting

Heart tissue samples were shredded using the tissue separator (IKA Ultra-Turrax; Sigma-Aldrich) in 1ml lysis buffer containing 20 mM Tris, 10 mM DTT, 500mM Sodium chloride, 1% NP40, 12,5% Glycerol. The protease inhibitor cocktail tablets (Roche) and phosphatase inhibitor cocktails 2 and 3 (Sigma-Aldrich) were added just before usage. NRVCM were lysed and scratched off the 6-well plate in 180μl lysis buffer. After 3 cycles of freeze-thaw, lysate was centrifuged at 10000xg to remove the cell debris. Protein concentration was measured by photometry using Bradford Protein Assay Kit (Bio-Rad). For immunoblotting protein samples were resolved by 10% SDS-PAGE and transferred to a polyvinylidene fluoride membrane (GE Healthcare). Membranes were blocked with 5% dry-milk in TBS-T for 2h and incubated with respective primary antibodies at 4°C overnight. After thorough washes with TBS-T buffer, secondary antibody containing horseradish peroxidase (HRP) for chemiluminescence or AF546 fluorophore for fluorescence was applied at room temperature for 2h. For detection, the ECL Select Western blotting detection reagents (GE Healthcare) was used and blots were analyzed with the FlourChem Q by Alpha Innotech. Primary antibodies used for western blotting are: anti-α‑actinin (Sigma, 1:5000), anti-Cofilin-2 (1:1000, Merck‑Millipore), anti-GAPDH (1:8000, Sigma‑Aldrich), anti-RhoA (1:1000, Cell Signaling), anti-SRF (1:1000, Cell Signaling), anti-α‑Tubulin (1:8000, Sigma‑Aldrich), and anti-Vimentin (1:500, Santa Cruz Biotechnology). Following secondary antibodies were used for western blotting: anti-mouse IgG-HRP (1:10,000, Santa Cruz Biotechnology), anti-rabbit IgG-HRP (1:10000, Santa Cruz Biotechnology) and anti-mouse IgG-AF546 (1:1000, Thermo Fisher Scientific).

### Histology

Mouse hearts were molded into Tissue-Tek Cryomolder (Sakura Finetek), and frozen on dry ice. Cryosections of 7 μm thickness were used for the histology. Lectin staining was carried out using FITC conjugated lectin from Triticum vulgaris (wheat), according to the manufacturer's instructions. Images were captured on BZ-9000 immunofluorescence microscope (Keyence) and cross-sectional area of the cardiomyocytes was analyzed with ImageJ software (version 1.46). The extent of fibrosis was measured by Sirius-red/fast green staining as described earlier [31, 32]. Images captured on BZ-9000 Keyence microscope were analyzed by BZ-II Analyzer software to measure the fibrotic area.

### Cloning of rat Cofilin-2

Rat Cofilin-2 was cloned using rat heart cDNA and Invitrogen™ Gateway® cloning technology (all Thermo Fisher Scientific). Primers used were, attB_Cofilin‑2_F: 5’-ggg gac aag ttt gta caa aaa agc agg ctt cga agg aga tag aac cat ggc atc tgg agt tac agt gaa tg-3’, attB_Cofilin‑2_R: 5’-ggg gac cac ttt gta caa gaa agc tgg gtc cta cag tgg ctt tcc ttc cag gga-3’. PCR product was recombined using BP Clonase™ II into the Gateway® pDONR™221 entry vector which upon sequence confirmation, transferred via LR Clonase™ II to Gateway® pAd/CMV/V5-DEST destination vectors, for transfection or transduction, respectively

### Isolation and culture of NRVCM and fibroblasts

NRVCMs were isolated as described before [31, 33]. In short, 1–2 days old Wistar rats (Charles River) were decapitated to obtain the hearts, which were stored in ice cold ADS buffer (120 mmol/L NaCl, 20 mmol/L HEPES, 8 mmol/L NaH2PO4, 6 mmol/L glucose, 5 mmol/L KCl and 0.8 mmol/L MgSO4 (pH 7.4)). The ventricles were minced with the scissor and digested 4–5 times in sterile ADS buffer containing collagenase type II (0.5 mg/mL, Worthington Biochemical Corporation) and pancreatin (0.6 mg/mL, Sigma-Aldrich) to separate the cells. By performing a gradient centrifugation using Percoll (GE Healthcare), NRVCM were purified and separated from cardiac fibroblasts. The NRVCM were incubated at 37°C in complete DMEM media (DMEM supplemented with 10% FCS, 100 U/mL penicillin, 100 μg/mL streptomycin and 2 mmol/L L-glutamine (Thermo Fisher Scientific)) for 24h before virus transduction or other downstream treatments. Fibroblast fraction from the gradient step mentioned above was plated in 6x well culture plates at 37°C for 4 h in complete DMEM medium, followed by media aspiration and washing with PBS to remove floating contaminant cells. Adhered fibroblasts were incubated for further 96 h in complete DMEM at 37°C.

### Cell surface area measurement

Immunofluorescence staining and cell surface area measurements were performed as detailed earlier [34]. Briefly, cardiomyocytes cultured on coverslips in 12x well culture plates, either transduced with Cfl2 overexpression/knockdown adenoviral particles, or transfected with miR-301a mimic/inhibitor, were washed 2x with PBS and fixed with 4% paraformaldehyde for 10 min. Fixed cells were washed 2x with PBS followed by a common step of permeabilization and blocking with 0.1% Triton X-100 in 2.5% BSA for 1 h at room temperature. Cells were then incubated for 1 h with primary anti–α-actinin antibody (1:200; Sigma-Aldrich), 5x washes with PBS, followed by the incubation with respective secondary antibody conjugated to Cy3 (Dianova) and DAPI for nuclear staining. After washings with PBS, coverslips were mounted on glass-slides using Fluoromount (Biozol). Immunofluorescence images were captured using BZ-9000 microscope (Keyence). Cell surface area was measured using HybridCellCount module BZ-II Analyzer software (Keyence).

### Transfection of miR-301/anti-miR301

NRVCM were transfected 24h post seeding using Lipofectamine® RNAiMAX (Thermo Fisher Scientific) to transfect microRNA miR-301a mimic/inhibitor, or a control microRNA, as per manufacturer's recommendation. Used microRNA mimics were: mirVana™ miRNA Mimic, Negative Control #1 and miR‑301a‑3p (both Thermo Fisher Scientific). Used microRNA inhibitors: miRCURY LNA Inhibitor Negative Control A and hsa‑miR‑301a (both Exiqon).

### Generation of miR-301a predicted binding site mutants and luciferase assay

Murine genomic DNA was used as a template for cloning 3’ untranslated regions (3’UTR) of the genes of interest into pmirGLO vector (Promega). Mutants of the predicted binding sites in the 3’UTR of Cofilin-2 were generated by QuikChange® II site-directed mutagenesis kit (Agilent Technologies) by replacing bases 2’-6’ of the seed sequence (5’GCACT‑3‘ to 5‘‑TACAG‑3). C2C12 cells (24-well format, 30.000 cells/well) were co-transfected with 20ng pmiRGLO vector per well and mimics (20pmol/well, mirVana™ miRNA Mimic, Negative Control #1 and miR‑301a‑3p, Thermo Fisher Scientific) 24h post cell seeding and incubated for additional 48h with one change of media. Cells were then lysed using passive lysis buffer provided with the Dual-Glo® Luciferase Assay System (Promega), and chemiluminescence was measured using the Infinite M200Pro (Tecan) in a 96-well format.

#### Primers used for 3’UTR cloning

Acvr1‑3‘-XbalI‑F 5’-act gtc tag acc ttg tca ccg gtg tca aga agg a-3’, Acvr1‑3’‑SalI‑R 5’-act ggt cga cga ctt gaa aac agt tta ttt aat tta tac-3’, Cfl2‑3’‑XholI‑F 5’-agc tct cga gaa taa tag cca agt gcc att tg-3’, Cfl2‑3’‑SbfI‑R 5’-agc tcc tgc agg cac tta taa ttt tgc aag cta gca g-3’, Clcn3‑3’-XbalI‑F, 5’-act gtc tag agt cct gta gat gag gac aga-3’, Clcn3‑3’SalI‑R, 5’-act ggt cga cca ctt tta agt aga gat ttt aat aga tc-3’, Qk‑3’-XholI‑F 5’-agc tct cga gct tgc tgg atg aag gac ta-3’, Qk ‑3’-SbfI‑F 5’-agc tcc tgc agg gct ttt tca att att cta ttt aca aac aac-3’

#### Primers used for site directed mutagenesis

Cofilin‑2_pos_370_F 5’-cag tat tat tta tag ttt aca gtg att acc gtt ctc tga ggc act gg-3’, Cofilin‑2_pos_370_R 5’-cca gtg cct cag aga acg gta atc act gta aac tat aaa taa tac tg-3’, Cofilin‑2_pos_890_F 5’-ggg taa cgg tga tta agc tct tac agg gta ttt gga att ttt ttt cc-3’, Cofilin‑2_pos_890_R 5’-gga aaa aaa att cca aat acc ctg taa gag ctt aat cac cgt tac cc-3, Cofilin‑2_pos_1030_F 5’-gga gat cag aaa aa aat tct ttt tta cag ttg gcc tat cca gta tc-3’, Cofilin‑2_pos_1030_R 5’-gat act gga tag gcc aac tgt aaa aaa gaa ttt ttt tct gat ctc c-3’, Cofilin‑2_pos_1717_F 5’-gac ttg gga tct ttt tat aca gaa gga att tga ttt cag cct tcc-3’, Cofilin‑2_pos_1717_R 5’-gga agg ctg aaa tca aat tcc ttc tgt ata aaa aga tcc caa gtc-3’

### SRF-reporter assay

The luciferase reporter assay was carried out in 24-well format in C2C12 Cells (ATCC®) or in 12-well format in NRVCMs. As an indicator for the serum response factor (SRF) activity the pGL4.34[luc2P/SRF‑RE/Hygro] vector by Promega (20ng/well) was used, which contains a SRF responsive element promotor prior to the firefly luciferase gene. For normalization, Renilla luciferase containing pGL4.74[hRluc/TK] vector by Promega (5ng/well) was used. These vectors were co-transfected with either Cofilin-2 or miR-301a knockdown (Negative Control siRNA, Qiagen®; Cofilin 2 siRNA (m): sc‑37026, Santa Cruz Biotechnology), or overexpression constructs (n = 6) using Lipofectamine® 2000 Transfection Reagent (Thermo Fisher Scientific) 24h after seeding the C2C12 cells. Cells were cultured for 48h, lysed and luciferase assay was performed as mentioned above for 3’UTR mutants. Reporter assays in the NRVCM were performed using SRF-RE driven firefly luciferase adenovirus construct as described in [32]. Adenovirus encoding Cfl2 or a synthetic microRNA specifically targeting Cfl2 was used for the overexpression or knockdown of Cfl2, respectively.

### Statistical analysis

The error bars represent the standard error of the mean (SEM), unless stated otherwise. The statistical analysis was carried out using either two-tailed student’s *t-test* when comparing two groups. Equal distribution of the cell size measurement data was tested by Shapiro–Wilk test, and samples were compared by Kruskal-Wallis test (one-way ANOVA on ranks). *P*-values ≤ 0.05 were considered as statistically significant.

## Results

### Calsarcin-1 deficient mouse in pure C57BL/6 genetic background displays dilated cardiomyopathy phenotype

**Fig 1:**
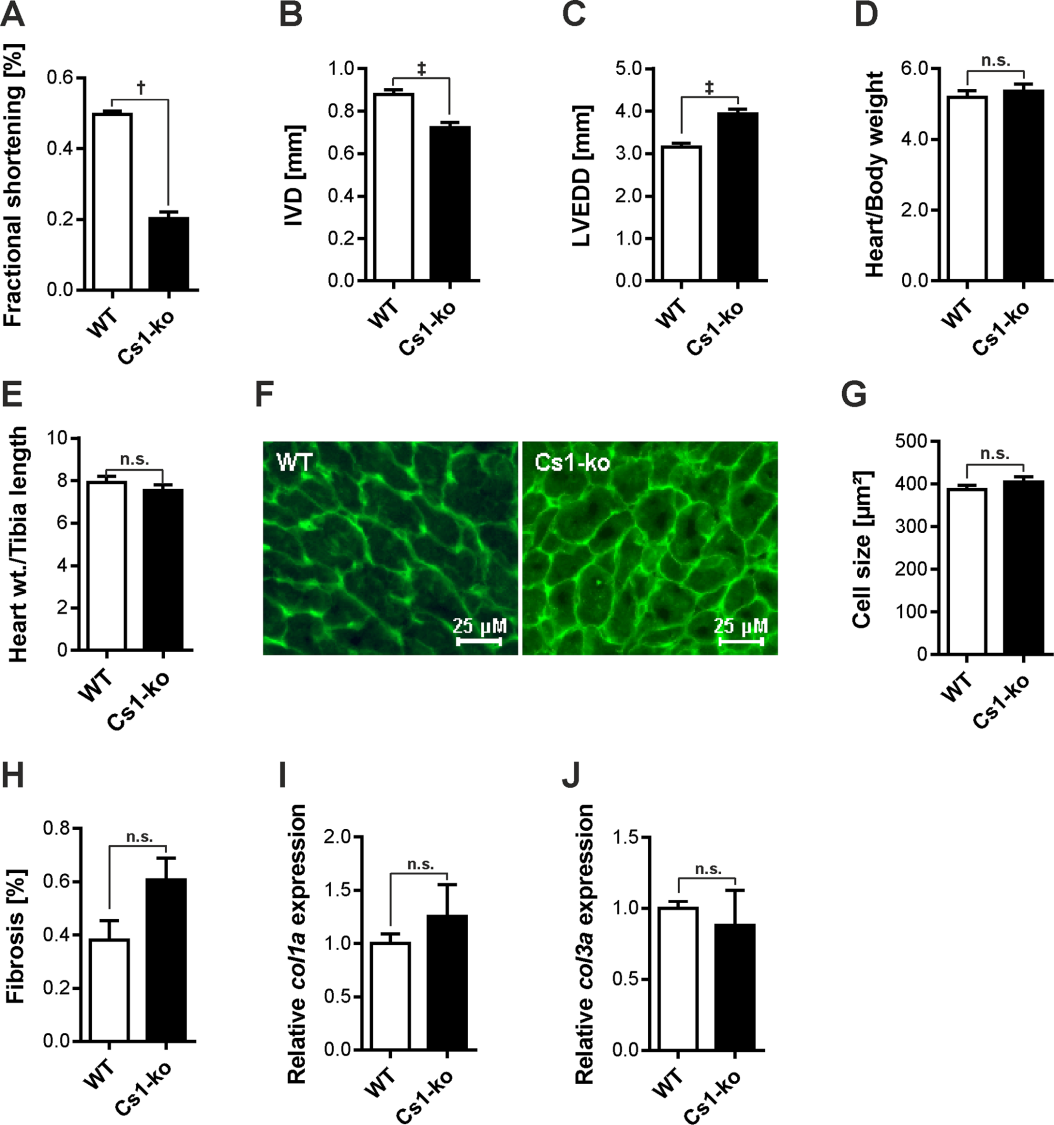
Phenotypic characterization of Calsarcin-1 deficient mice in C57BL/6 background. 13 weeks old mice underwent echocardiography to assess cardiac function. Reduced fractional shortening (**A**), intraventricular diameter (IVD, **B**), and increased left ventricular end diastolic diameter (LVEDD, **C**) indicates contractile dysfunction and cardiac dilatation of Calsarcin-1 deficient mice (Cs1-ko) compared to wild-type (WT) littermates. Ratios of heart weight to body weight (**D**), and heart weight to tibia length (**E**) were unchanged between both genotypes suggesting no cardiac hypertrophy (N = 7 (WT), and 10 (Cs1-ko) for A-E). In line with unchanged heart weights, there was no difference in cardiomyocyte cell size in Cs1-ko or WT mice as determined by lectin staining (**F**), and measurement of cell surface area (**G**) (N >150 for G). (**H**) Bar graph indicating no difference in the fibrotic lesions in Cs1-ko mice compared to wild-type littermates (N = 3 each). Similarly, there was no difference in the expression levels of collagen I (**I**) and III (**J**), determined by quantitative real-time PCR. The statistical analysis was carried out using two-tailed student’s *t-test*. *: p<0.05, †: p<0.01, ‡: p<0.001, n.s.: non-significant.

We have earlier reported that mice with Calsarcin-1 null mutation in a mixed genetic background do not exhibit any overt basal cardiac hypertrophy phenotype, however show accelerated hypertrophic cardiomyopathy in response to pathological biomechanical stress [13]. We then back-crossed these mice for more than 10 generations to obtain Calsarcin-1 deficiency in a pure C57BL/6 genetic background (henceforth these mice will be referred as Cs1-ko). Using echocardiography analysis we found that Cs1-ko mice showed severely reduced fractional shortening (Fig 1A) and intra-ventricular diameter (Fig 1B), whereas, left ventricular end-diastolic diameter was significantly increased (Fig 1C). Surprisingly however, there was no difference between heart weight to body weight (Fig 1D), and heart weight to tibia length ratios (Fig 1E) in Cs1-ko mice. Moreover, in line with heart weight to body weight ratios, cardiomyocyte cell surface area was also unaltered between both the genotypes (Fig 1F and 1G). Furthermore, lack of Calsarcin-1 did not increase fibrosis as evident from the unaltered fibrosis and unchanged expression of fibrosis markers, Collagen I and III (Fig 1H–1J). Altogether, these data suggests that Cs1-ko mice in pure C57BL/6 background displays strict dilated cardiomyopathy phenotype.

### MicroRNA miR-301a is downregulated in Cs1-ko mice

**Fig 2:**
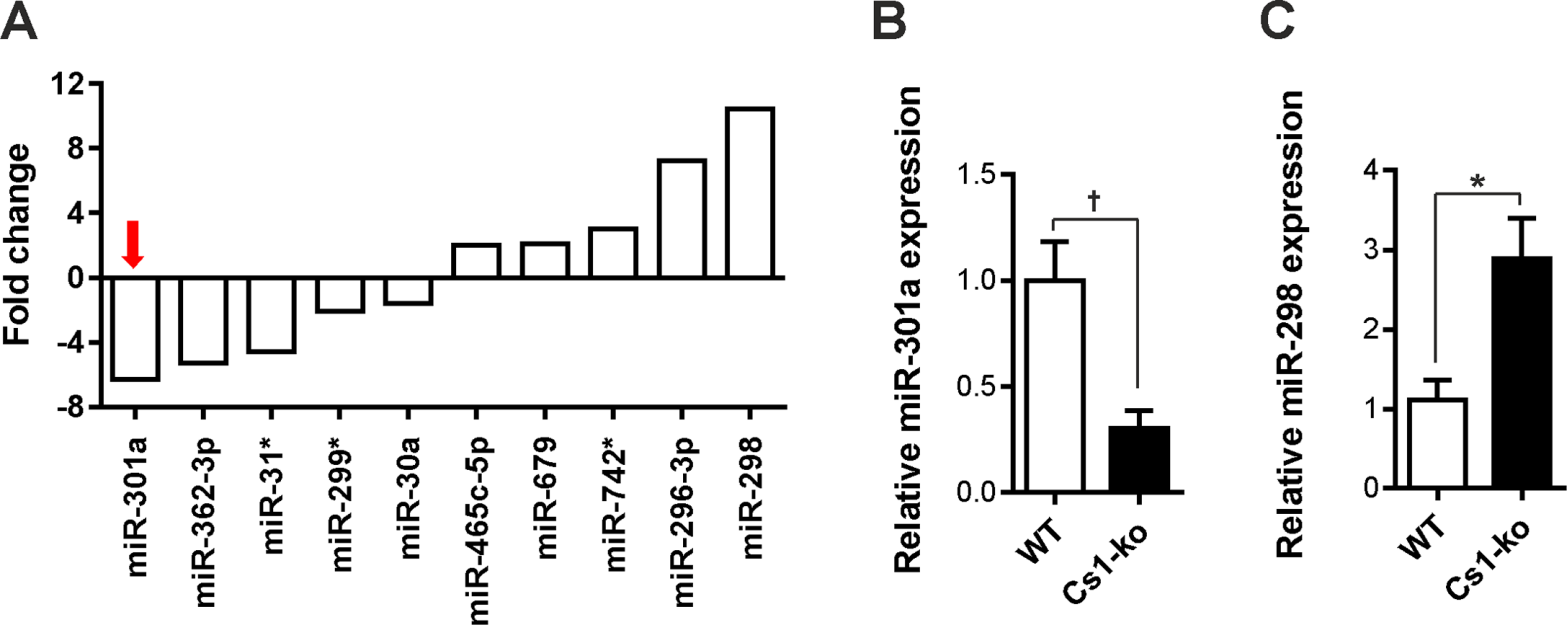
MicroRNA miR-301a is downregulated in Cs1-ko mice. Microarray analyses were performed on Illumina Mouse Sentrix-6 BeadChip arrays (Illumina, Inc.) using total RNA isolated from Calsarcin knockout (Cs1-ko) and wild-type (WT) mice. Microarray scanning was done using a Beadstation array scanner and analyzed by normalization of the signals using the quantile normalization algorithm without background subtraction. Differentially regulated microRNAs were defined by calculating the standard deviation differences of a given probe in Cs1-ko and WT genotypes. (**A**) Bar graph presenting few selected dysregulated microRNAs in Cs1-ko mice compared to WT mice. MiR-301a was identified the most downregulated microRNA, whereas, miR-298 was highly upregulated (N = 4 each), which was confirmed in independent cohort by quantitative real-time PCR for miR-301a (**B**), and miR-298 (**C**) (N = 5 (WT), and 6 (Cs1-ko)). Statistical analysis was carried out using two-tailed student’s *t-test*. *: p<0.05, †: p<0.01.

To understand the molecular causes of dilated cardiomyopathy phenotype of Cs1-ko mice, we performed comparative microRNA microarray analysis of Cs1-ko with wild-type mouse heart. Microarray data revealed that several microRNAs were dysregulated in Cs1-ko mice (S1 Table), including miR301a, which was maximally downregulated, whereas, miR-298 was highly upregulated in Cs1-ko mouse hearts (schematically depicted in Fig 2A). We further validated the expression of miR-301a and miR-298 by quantitative real-time PCR (qPCR) in independent set of mouse cohort to confirm its downregulation (Fig 2B and 2C).

### miR-301a targets Cofilin-2 in cardiomyocytes and in Cs1-ko mice

**Fig 3:**
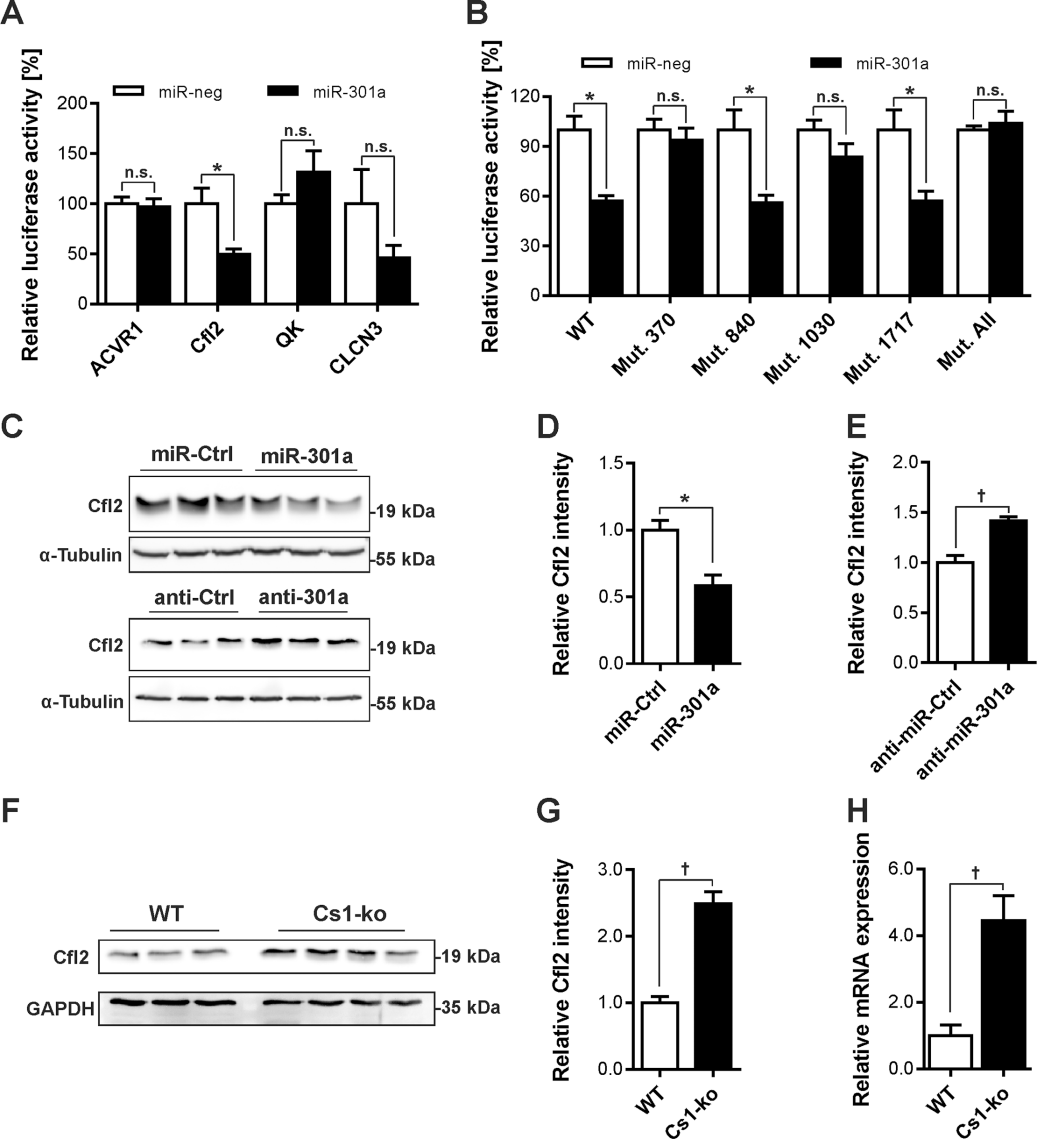
Cofilin-2 is a target of miR-301a. (**A**) We selected a subset of the targets identified through online microRNA target database search, including Cofilin-2 (Cfl2), Activin A Receptor Type 1 (ACVR1), Quaking (Qk), and Chloride Voltage-Gated Channel 3 (CLCN3). Putative 3’UTR binding sites from these targets was evaluated using pmirGLO Dual-Luciferase miRNA Target Expression Vector and assay system and found that only Cfl2 was a possible target (N = 6). (**B**) Cfl2 3’UTR has 4 putative binding sites (detailed in S1A–S1D Fig), which we mutated using site directed mutagenesis to confirm which of these binding sites act as targets for miR-301a binding. Mutations in binding sites 370 and 1030 resulted in loss of luciferase activation, clearly suggesting that these two sites act as binding sites for miR-301a (N = 4). (**C**) Immunoblot showing that the overexpression of miR-301a mimic downregulated (N = 3 each, original uncropped blots are shown in S1B & S1C Fig, respective densitometry is shown as a bar graph in **D**), whereas, overexpression of miR-301a inhibitor upregulated (respective densitometry is shown as a bar graph in **E**) the protein levels of Cfl2. (**F**) Cfl2 was found upregulated in Cs1-ko mice as depicted in an immunoblot at protein (N = 3 (WT), 4 (Cs1-ko), original uncropped blots are shown in S1D Fig, respective densitometry is shown as a bar graph in **G**), and at transcript level determined by immunoblotting and quantitative real-time PCR (**H**), respectively. All experiments are repeated at least two times and the statistical analysis was carried out using two-tailed student’s *t-test*. *: p<0.05, †: p<0.01, n.s.: non-significant.

Next, we used online prediction databases to identify possible miR-301a targets which resulted into hundreds of putative targets. We selected few of the targets including Cofilin-2 (Cfl2), Activin A Receptor Type 1 (ACVR1), Quaking (Qk), and Chloride Voltage-Gated Channel-3 (CLCN3) for further validation using pmirGLO Dual-Luciferase miRNA Target Expression Vector and assay system. Luciferase activity was found to be reduced only in Cfl2 construct (Fig 3A) which led us evaluate its possible miR-301a binding sites in details. We found four putative miR-301a binding sites in 3’UTR of Cfl2 depicted in S1A Fig which we mutated by site directed mutagenesis and studied for the validation of miR-301a binding. Mutation in two of the four putative binding sites (binding sites at position 370 and 1030 of the 3’UTR) prevented the reduction in luciferase activity by miR-301a overexpression suggesting that these two binding sites are responsible for miR-301a effect on Cfl2 expression (Fig 3B). We then confirmed if miR-301a targets Cfl2 *in vitro* in neonatal rat ventricular cardiomyocytes (NRVCM). As anticipated, overexpression of miR-301a reduced while its knockdown increased Cfl2 expression determined by immunoblotting (Fig 3C–3E). Finally, we found a strong *in vivo* correlation between downregulation of miR-301a to the upregulation of Cfl2 in Cs1-ko mice (Fig 3F–3H), suggesting a possible physiological importance of miR-301a in regulating Cfl2 in the heart.

### Expression of miR-301a and Cfl2 is higher in cardiomyocytes than fibroblasts

**Fig 4:**
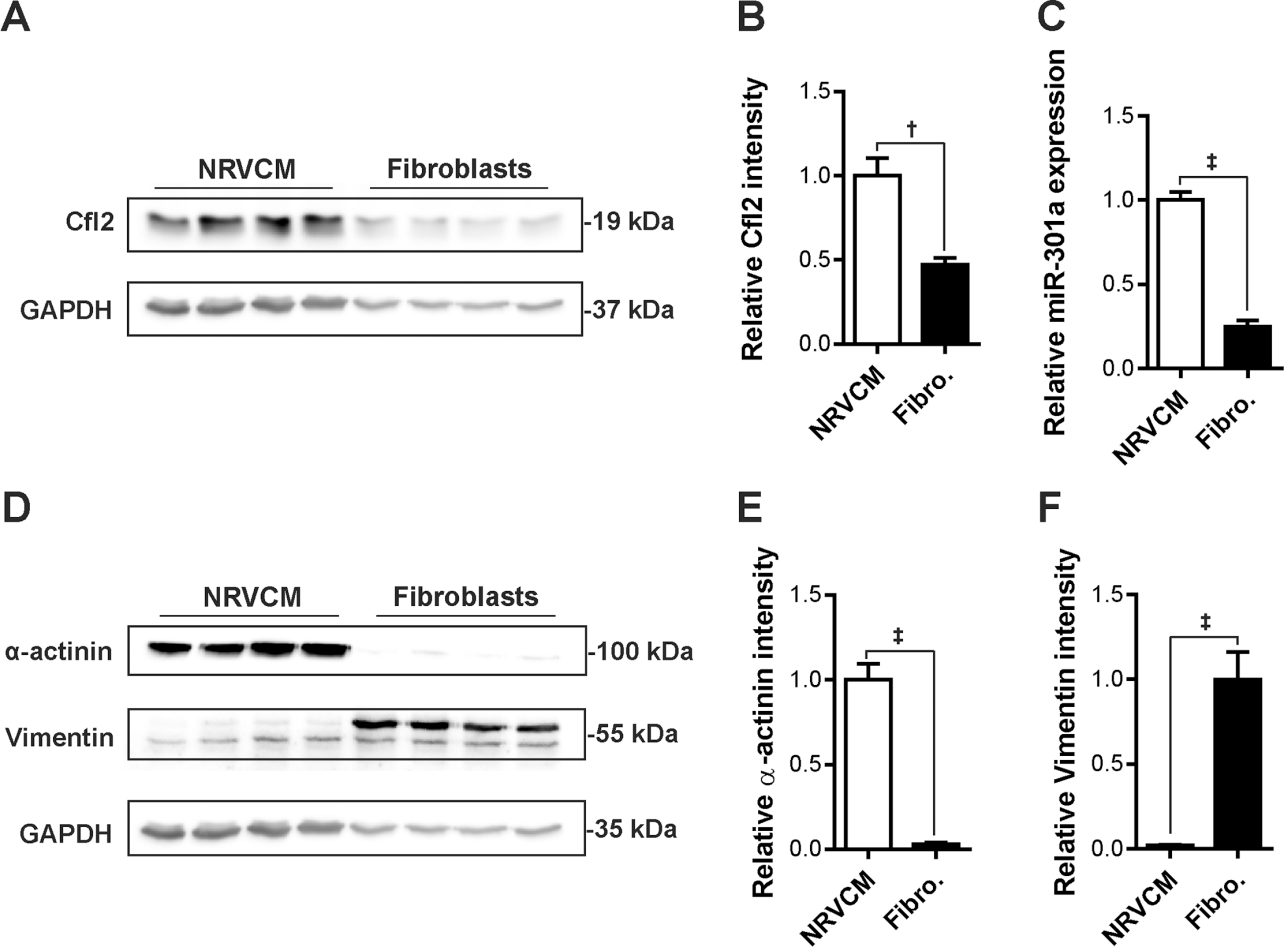
Expression of miR-301a and Cfl2 is higher in cardiomyocytes compared to fibroblasts. Protein and RNA was extracted from isolated neonatal rat cardiomyocytes and fibroblasts for comparative analysis of miR-301a and Cfl2 expression status. (**A**) Expression levels of Cfl2 is found to be higher in cardiomyocytes compared to the fibroblasts shown in an immunoblot (original uncropped blots are shown in S2B Fig, respective densitometry is shown as a bar graph in **B**). (**C**) Expression of miR-301a was also higher in cardiomyocytes than fibroblasts as determined by quantitative real-time PCR. (**D**) α-actinin and vimentin levels were determined in the same samples as markers of cardiomyocytes and fibroblasts, respectively, to assess the purity of isolated cells (original uncropped blots are shown in S2C Fig, respective densitometry is shown as a bar graph in **E, F**). N = 4 each. The statistical analysis was carried out using two-tailed student’s *t-test*. †: p<0.01, ‡: p<0.001.

Tissue distribution pattern for miR-301a determined by qPCR revealed its ubiquitous expression in the heart, brain, skeletal muscle, etc. (S2 Fig). To discriminate the expression of miR-301a and Cfl2 in cardiac major cell types, we studied their expression levels in isolated fibroblasts and cardiomyocytes from neonatal rat ventricles. We found that the expression of both miR-301a and Cfl2 was higher (>2 fold) in cardiomyocytes compared to fibroblasts (Fig 4A–4C). Expression of α-actinin and vimentin were used as markers to determine the purity of cardiomyocytes and fibroblasts, respectively (Fig 4D–4F).

### miR-301a and Cfl2 oppositely regulates Rho-mediated SRF signaling but not cellular hypertrophy

**Fig 5:**
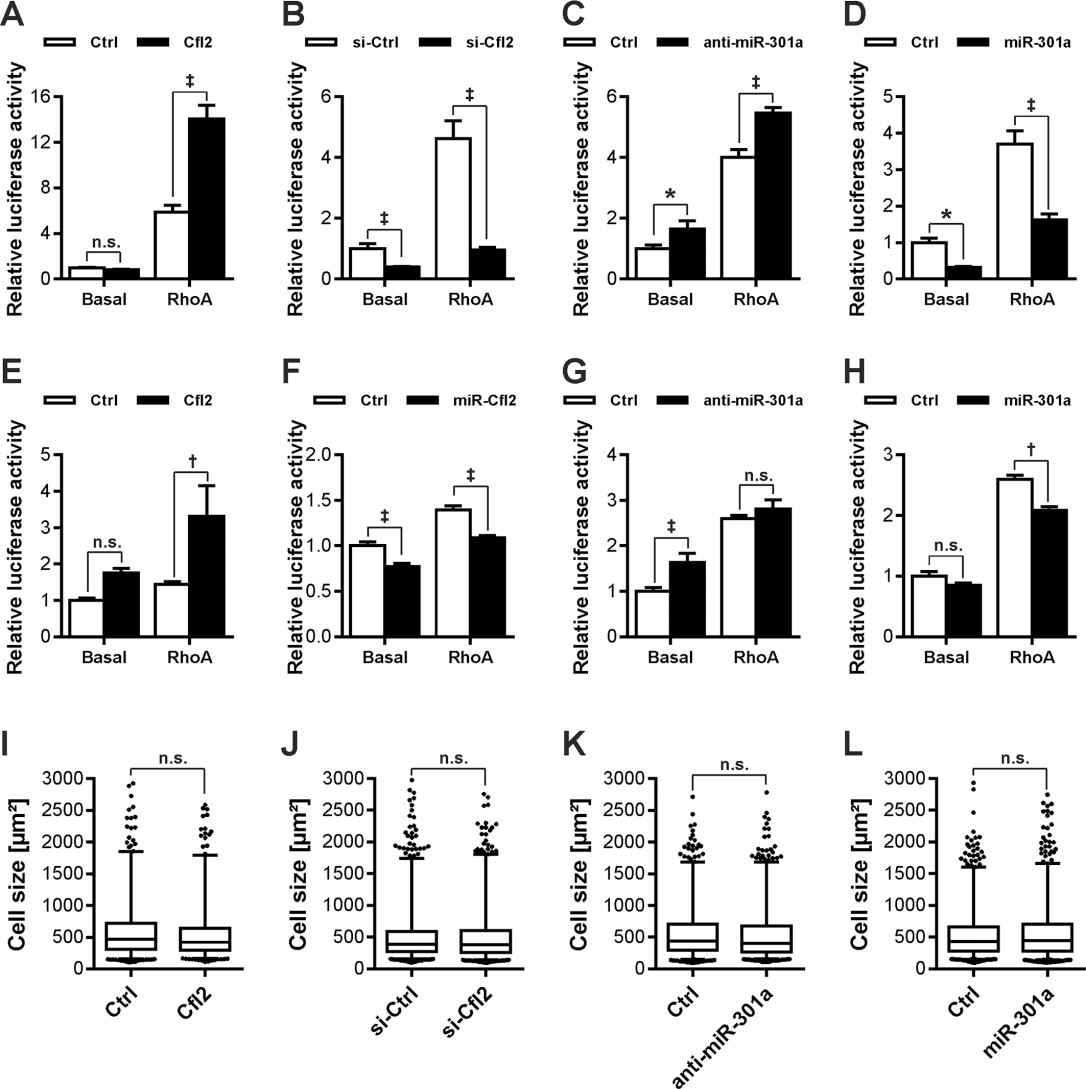
miR-301a and Cfl2 oppositely regulates Rho-mediated SRF signaling. Cfl2 or miR-301a were either overexpressed or knocked-down using respective vectors, mimic, or inhibitor transfection in C2C12 cells together with a luciferase construct carrying SRF-RE driven firefly luciferase. Overexpression of Cfl2 additively increased the luciferase activation by either constitutively RhoA (**A**), whereas, its siRNA led to inhibition of luciferase activation, basal, as well as in presence of RhoA (**B**). Knockdown of miR-301a also increased the luciferase activation (**C**), and the overexpression of miR-301a mimic significantly blunted the luciferase activity (**D**). For SRF-gene reporter assays in NRVCM, Cfl2 was overexpressed using adenovirus encoding rat Cfl2, whereas, its knockdown was achieved using adenovirus encoding synthetic microRNA specifically targeting Cfl2. Expression of miR-301a was modulated in NRVCM same as in C2C12 cells. Like in C2C12 cells, Overexpression of Cfl2 in NRVCM also exhibited positive effect on the activation of luciferase activity (**E**); knockdown of Cfl2 on the other hand significantly inhibited the activation of SRF-RE driven luciferase activity (**F**). Consistently, altered expression of miR-301a by treating NRVCM with miR-301a inhibitor (**G**) or mimic (**H**) oppositely affected the luciferase activation. Increased expression of Cfl2 in NRVCM did not alter cell surface area (**I**). Knockdown of miR-301a also led to no effect on cell size (**J**). Similarly, siRNA mediated knockdown of Cfl2 (**K**) or overexpression of miR-301a too did not alter cell surface area (**L**). N>500 for cell size measurements, and N = 6 luciferase assays. All experiments have been repeated at least twice. The statistical analysis was carried out using two-tailed student’s *t-test*. *: p<0.05, ‡: p<0.001, n.s.: non-significant.

The ADF/cofilin family proteins are actin-binding that are actively involved in actin remodeling. RhoA, a small Rho family GTPase and an activator of serum response factor (SRF) signaling has also been shown to play an essential role in the control of myocardial fibrosis by regulating cofilins [35]. We therefore hypothesized that Cfl2 plays an essential role in RhoA-SRF activation and miR-301a will negatively affect this activation by regulating Cfl2 expression. To test this hypothesis, we performed SRF-response element (SRF-RE) driven firefly luciferase activity assay by either overexpressing or knocking down Cfl2/miR-301a in C2C12 cells. Overexpression of Cfl2 alone did not regulate the luciferase activity (Fig 5A). Surprisingly however, co-expression of Cfl2 and RhoA (both constitutively active and native RhoA) dramatically increased the activation of luciferase reporter (Fig 5A). In contrast, knockdown of Cfl2 strongly attenuated the luciferase activity not only at basal level, but also inhibited the RhoA-mediated SRF-RE activation (Fig 5B) suggesting that Cfl2 is necessary and sufficient for the SRF-signaling activation through RhoA. In contrast, downregulation of miR-301a resulted in the activation, whereas, its overexpression significantly blunted the basal as well as RhoA-mediated activation of SRF-RE signaling (Fig 5C and 5D). Importantly, strong effect on RhoA-mediated SRF activation via Cfl2 overexpression observed in C2C12 cells was consistent in cardiomyocytes as well (Fig 5E). Knockdown of Cfl2 also significantly blunted the activation of luciferase activity, both at baseline as well as in the presence of RhoA (Fig 5F). Similarly, inhibition of miR-301a expression effectively accelerated SRF-signaling at basal level (Fig 5G), whereas, its overexpression significantly abrogated the RhoA-mediated activation of SRF activity (Fig 5H). Finally, we evaluated if Cfl2/miR-301a influences cellular hypertrophy by measuring cell surface area. To our surprise, neither overexpression nor knockdown of Cfl2 or miR-301a affected the cell size in neonatal rat cardiomyocytes (Fig 5I–5L).

### Calsarcin-1 deficiency upregulates SRF in mouse heart

**Fig 6:**
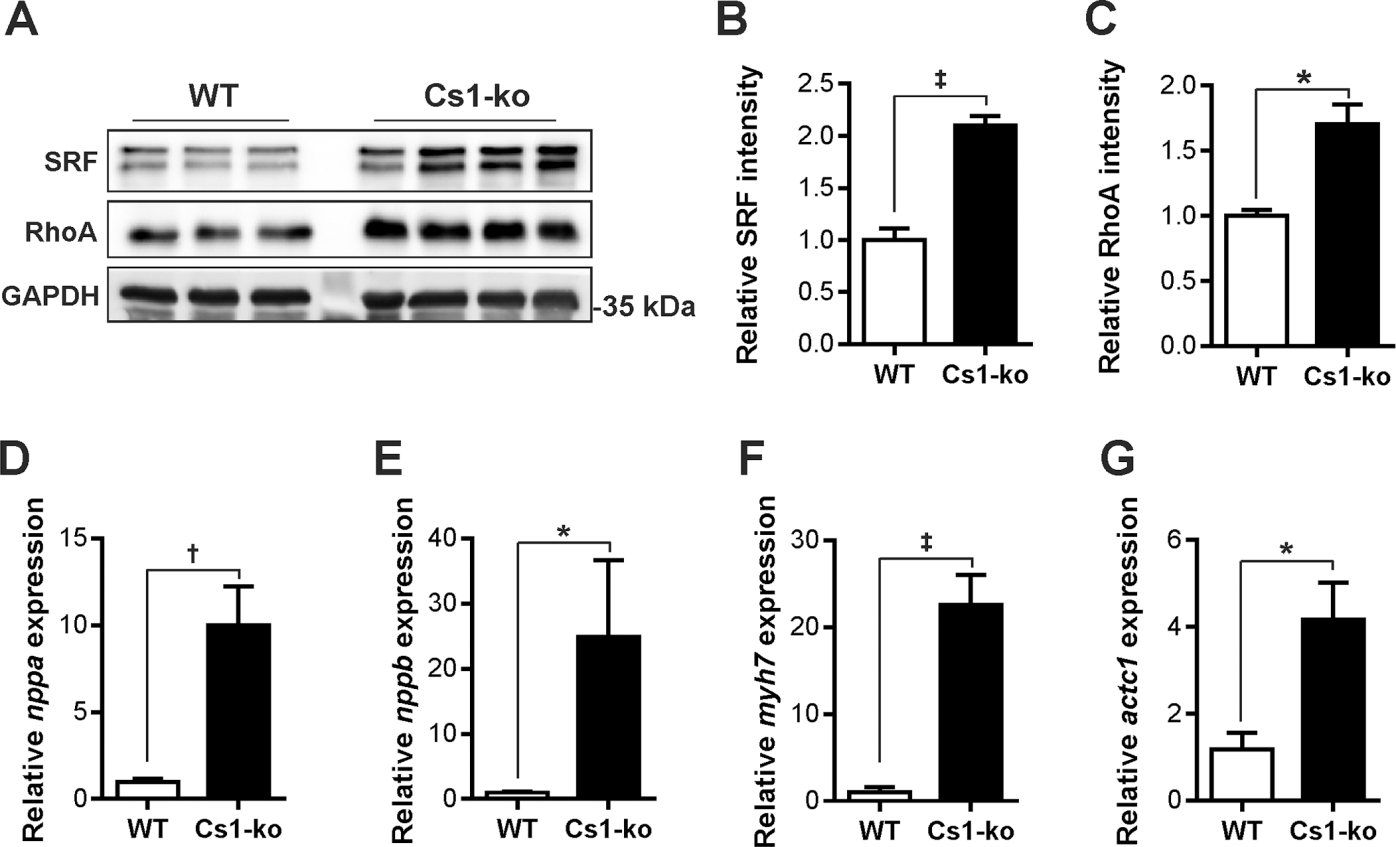
Calsarcin-1 deficiency upregulates SRF in mouse heart. (**A**) SRF and RhoA are upregulated in Cs1-ko mice as depicted in an immunoblot (N = 3 (WT), 4 (Cs1-ko), original uncropped blots are shown in S3 Fig, respective densitometries are shown as bar graphs in **B** and **C**). Fetal gene program was strikingly activated in Cs1-ko mice as detected by increased expression of *nppa* (**D**), *nppb* (**E**), and *myh7* (**F**). Similarly cardiac muscle α-actin (*actc1*) was highly upregulated in Cs1-ko mice. N = 5 (WT) and 6 (Cs1-ko). The statistical analysis was carried out using two-tailed student’s *t-test*. *: p<0.05, †: p<0.01, ‡: p<0.001.

Due to the significant effect of Cfl2 and miR-301a on RhoA/SRF-signaling in C2C12 cells/NRVCM, and observed upregulation of Cfl2 and downregulation of miR-301a in Cs1-ko mice, we determined the SRF and RhoA levels in Cs1-ko mice. We found considerable increase in the expression of both SRF and RhoA in Cs1-ko mice compared to the respective wild-type littermates (Fig 6A–6C). Increased levels of SRF in the heart is known to cause robust activation of SRF signaling, and in the absence of other stimuli, SRF upregulation is sufficient to cause cardiomyopathy [17]. Along these lines, the expression of fetal genes *nppa*, *nppb*, and *myh7*, which are also direct targets of SRF transcription factor were dramatically increased in the heart of Cs1-ko mice (Fig 6D–6F). Furthermore, *actc1* was also highly upregulated (Fig 6G), which is a bona fide target of SRF. Taken together, these data suggests an increased activation of SRF-signaling in Cs1-ko mice.

## Discussion

Cofilin-2, a member of the ADF/cofilins family proteins that regulates actin dynamics, is essential for maintaining actin filament length in muscle sarcomere [26]. Through a microRNA microarray we identified miR-301a is significantly downregulated in Calsarcin-1 deficient mice that present DCM phenotype in a pure C57BL/6 background. Functional characterization of this microRNA in cardiac perspective revealed that miR-301a targets and regulates Cfl2 *in vitro* in neonatal rat cardiomyocytes, and *in vivo* in Cs1-ko mice. Furthermore, our *in vitro* data indicated that miR-301a attenuates RhoA-mediated activation of SRF signaling via targeting Cfl2 *in vitro* without affecting cellular hypertrophy. Importantly, RhoA, SRF and its target genes were strongly upregulated in Cs1-ko mice where Cfl2 is upregulated and miR-301a is downregulated, suggesting a possible involvement of Cfl2 in SRF activation *in vivo*.

Natural genetic diversity in human population determines the extent of disease phenotype, including cardiac diseases, suggesting that genetic background also plays an important role in pathology. Similarly, increasing number of studies suggests the importance of selecting an appropriate mouse genetic background for cardiac evaluations [36, 37]. For example, ApoE knockout mouse displays severe atherosclerotic phenotype in C57BL/6 compared to FVB/J, whereas, MLP knockout has dramatically increased heart failure rate in the 129/Sv than C57BL/6 genetic background [38, 39]. Similarly, Lygate et al. ascertained that mitochondrial creatine kinase knockout mice do not display any cardiac phenotype in pure C57BL/6 genetic background [40], which earlier reportedly led to LV dysfunction and hypertrophy [41]. In congruence with these reports, we previously found that Calsarcin-1 knockout mice do not exhibit cardiac hypertrophy phenotype at baseline in mixed genetic background notwithstanding striking upregulation of fetal genes and contractile dysfunction; however, these mice displayed strict DCM phenotype, devoid of hypertrophy, even when back-crossed for more than 10 generations to obtain the desired mutation in pure C57BL/6 background. Therefore, to identify the molecular causes behind DCM phenotype despite lack of hypertrophy, we performed microRNA microarray which resulted in identification of several microRNAs that were differentially regulated in Cs1-ko mice.

MicroRNAs play crucial role in cardiac regeneration, energy homeostasis, and regulating cardiac physiology by targeting and fine-tuning the expression of several important transcription factors, cytoskeletal proteins, etc. [42–47]. Therefore, dysregulation of (a) microRNA(s) due to extrinsic and/or intrinsic stress further adds to the severity of the disease [48–50]. This very fact can and is being capitalized for the potential therapeutic uses of microRNAs against cardiac diseases [43, 48, 51–55]. Present study is conceived to identify the dysregulated microRNA(s), in Cs1-ko mice that developed dilated cardiomyopathy phenotype. Our microarray analysis revealed deregulation of many microRNAs (S1 Table). Few of the highly upregulated microRNAs were: miR-79, miR-183, miR-206, miR-207, miR-296-3p, miR-298, miR-380-5p, miR-433, miR-449b, miR-705, miR-761 (S1 Table). MiR-206 and miR-298 are previously shown to be upregulated in rat model of post-infarction heart failure [50]. However, circulating levels of miR-296 and miR-433 were found to be downregulated in hypertension and congenital heart disease, respectively [56, 57]. MicroRNAs like miR-19a, 34b, 129, 135a, 142-3p, miR-153, miR-186, miR-187, and miR-301a were significantly downregulated in Cs1-ko mice. Zhu et al. has recently demonstrated that increased expression of miR-135a, protects diabetic mice against ischemia/reperfusion injury [58]. Circulating levels of miR-129 and miR-142 were reduced in congestive heart failure [59, 60]. Majority of the dysregulated microRNAs e.g. miR-380, miR-207, miR-79, miR-129, miR-153, miR-183, etc. identified through our microarray analysis have not been associated with any cardiac anomaly yet, which can be characterized further for their potential role in the heart.

MiR-301a, the most-downregulated microRNA in our screen has previously been associated strongly with many human cancers including prostate cancer, malignant melanoma, osteosarcoma, etc. [61–64]. Although miR-301a is significantly expressed in the heart and other tissues, no cardiac role of this ubiquitously expressed microRNA is known yet. Moreover, we here found that miR-301a was highly expressed in isolated cardiomyocyte compared to fibroblasts, suggesting a cell-type specific function for this microRNA. Most interestingly, we discovered Cofilin-2 as one of the putative targets of miR-301a through microRNA target database search, which we further validated through series of experiments including luciferase assays, site directed mutagenesis of possible binding sites, and by manipulation of miR-301a expressions in neonatal rat cardiomyocytes. To strengthen these *in vitro* findings, we found a strong inverse correlation between Cfl2 and miR-301a expression in Cs1-ko mice.

**Fig 7:**
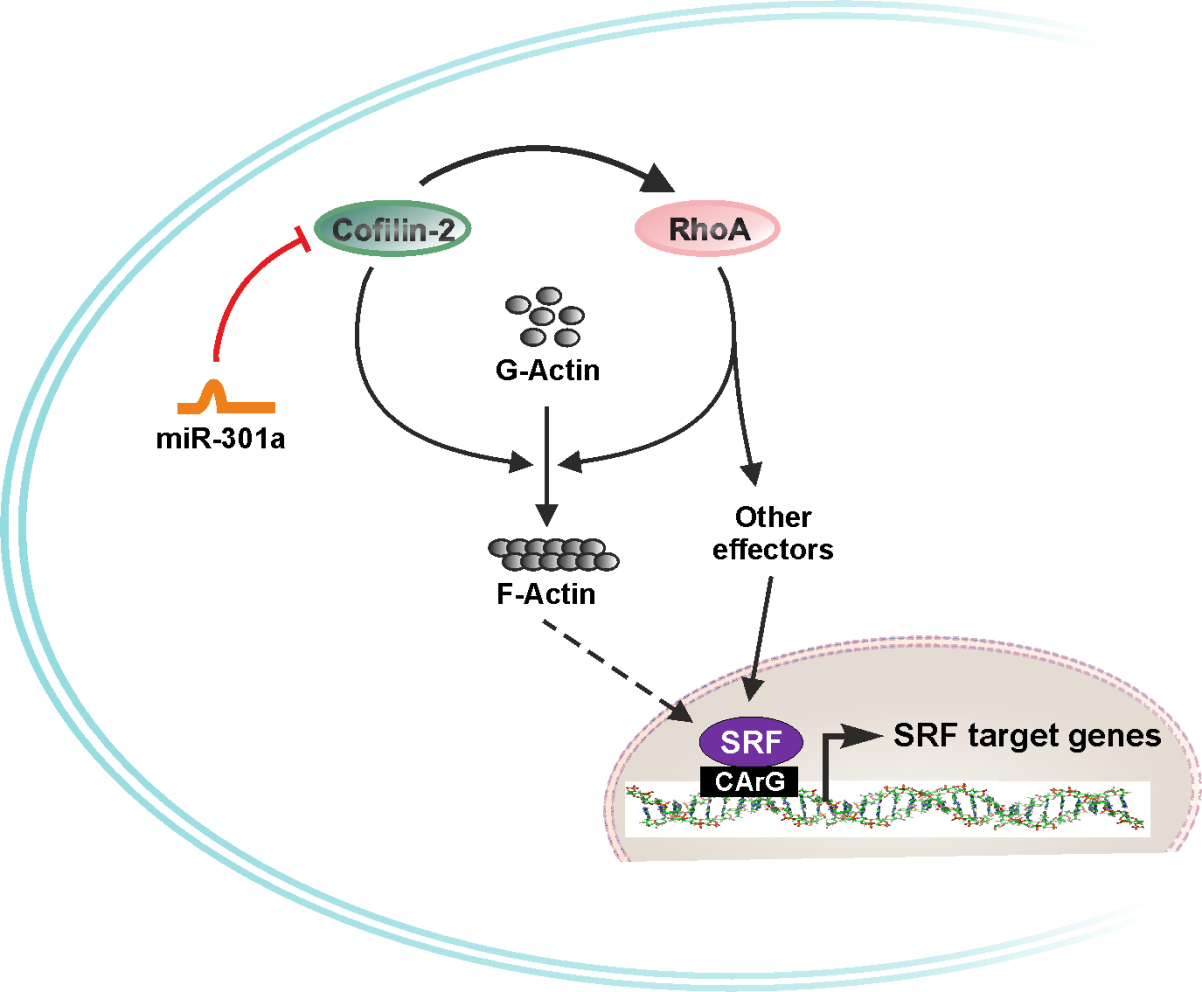
Model figure of cardiac role of Cfl2 and miR-301a. Our data from the present study illustrated involvement of Cfl2 in the activation of SRF signaling via RhoA. Both RhoA and Cfl2 plays important role in actin treadmilling. Increased levels of F-actin in turn activates SRF signaling. RhoA also activates SRF signaling through other effector molecules like myocardin-related transcription factors, etc.. On the other hand, miR-301a targets Cfl2 in cardiomyocytes, thereby inhibiting the activation of SRF signaling.

Cfl2 belongs to the family of actin severing proteins, primarily expressed in muscle, and also in the brain and liver [23], and is critical for the maintenance of sarcomeric actin dynamics and length [26, 27]. However, our data indicates that Cfl2 overexpression does not significantly alter the cell surface area of isolated NRVCM. Ablation of miR-301a resulted into similar effects as observed with the Cfl2 overexpression, pertaining to the increased levels of Cfl2 upon miR-301a knockdown. We also found that Cfl2 increases the RhoA-mediated SRF activation, whereas, miR-301a upregulation is sufficient to antagonize these effects (Fig 7). Moreover, increased expression of SRF/RhoA in Cs1-ko mice and target genes of SRF suggesting activation of SRF signaling in these mice. Of note, Cs1-ko mice do not exhibit any signs of hypertrophy notwithstanding upregulation of fetal gene program and contractile dysfunction. Similar findings were observed when we modulated the expression of Cfl2 or miR-301a. Overexpression of Cfl2 or knockdown of miR-301a though resulted in the activation of SRF signaling, neither of these treatments caused hypertrophy. These similar *in vitro* findings points towards possible involvement of Cfl2-miR301a in the pathophysiology of Cs1-ko mice, at least partially. Recently, increased expression and phosphorylation of Cfl2 has been linked with DCM and myocardial aggregates [28]. Surprisingly, deletion of Cfl2 in mice also led to progressive muscle degeneration and appearance of sarcoplasmic protein aggregates [65]. Both these findings highlight the importance of Cfl2 in muscle and heart pathophysiology. Here, we show that miR-301a regulates the expression as well as physiological function of Cfl2 in cultured cardiomyocytes, which needs additional *in vivo* evaluations and validation by gain- and loss-of-function studies. We therefore propose to explore the possibility of use of miR-301a manipulations for therapeutic intervention to target cardiac disorders caused due to deregulation of Cfl2.

## Supporting information

S1 Fig Putative miR-301a binding sites in 3’UTR of Cfl2. Cofilin 3’UTR contains four possible miR-301a binding sites named as 370, 890, 1030, and 1717 as presented pictorially in **A**. Original uncropped blots are shown for Fig 3C (**B**, **C**), and 3F (**D**).
(DOCX)

S2 Fig Tissue distribution of miR-301a. (**A**) Expression of miR-301a was determined in various tissues by quantitative real-time PCR indicates ubiquitous distribution of miR-301a, including significant expression in the heart (N = 3). Original uncropped blots are shown for Fig 4A (**B**), and 4D (**C**).
(DOCX)

S3 Fig Original uncropped blots are shown for Fig 6A. (DOCX)

S1 Table Comparative microRNA microarray analysis data between Calsarcin-1 knockout and wild-type mice. (XLSX)
