# Supplementary material for: MicroRNA miR-301a is a novel cardiac regulator of Cofilin-2
Source: PLoS One. 2017 Sep 8;12(9):e0183901. doi: 10.1371/journal.pone.0183901 (PMC5590826; doi:10.1371/journal.pone.0183901)
Supplement: S1 Fig — Cofilin 3’UTR contains four possible miR-301a binding sites named as 370, 890, 1030, and 1717 as presented pictorially in A. Original uncropped blots are shown for Fig 3C (B, C), and 3F (D). (DOCX) [file pone.0183901.s001.docx]

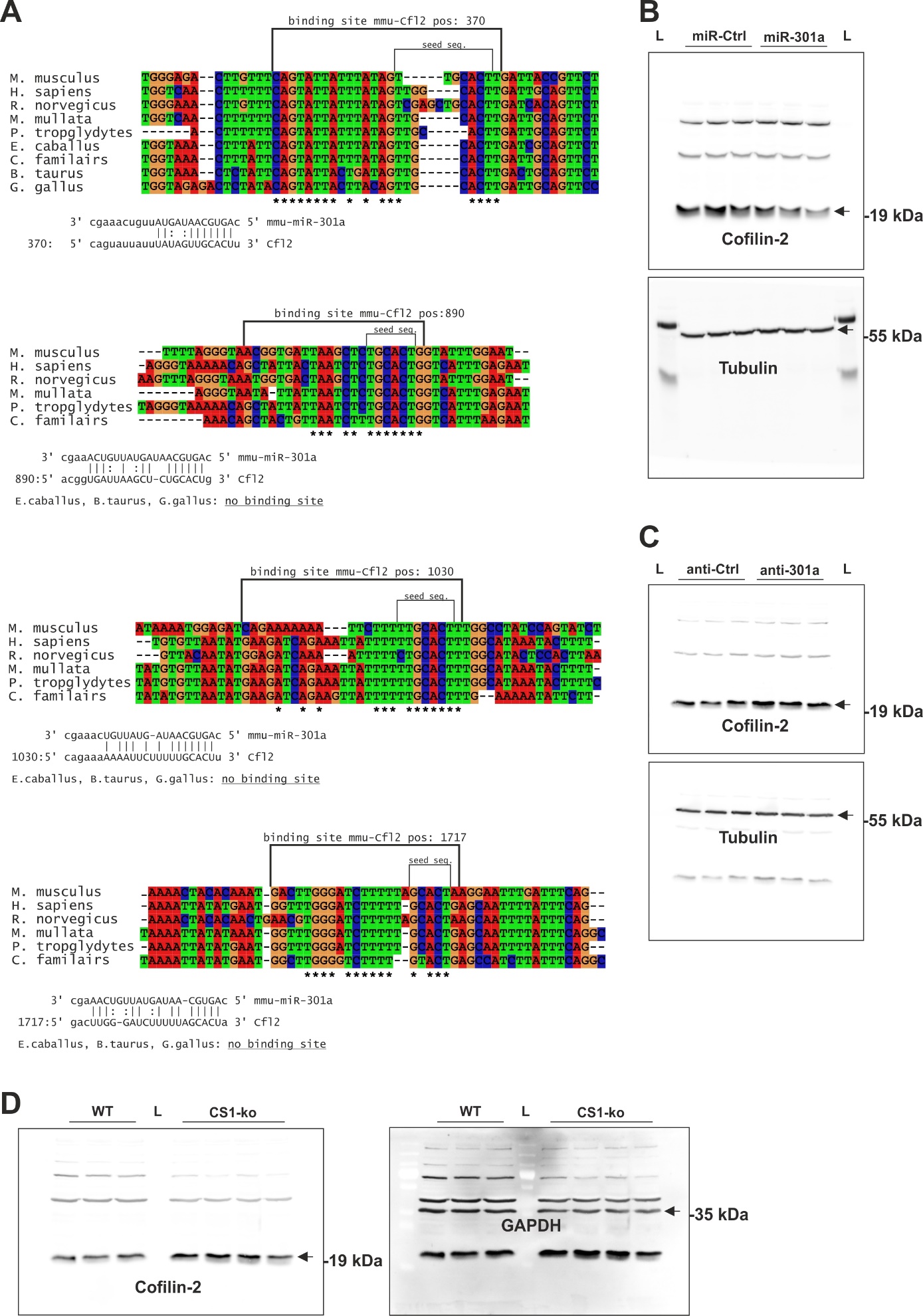


**Supplementary Fig. 1.** (**A**) Alignment of four putative miR-301a binding sites identified in 3’ UTR of Cofilin-2 gene from various vertebrates indicating the conserved binding region. Original uncropped blots for Figure 3C (**B**, **C**), and 3F (**D**). L, protein ladder
