# Supplementary material for: MicroRNA miR-301a is a novel cardiac regulator of Cofilin-2
Source: PLoS One. 2017 Sep 8;12(9):e0183901. doi: 10.1371/journal.pone.0183901 (PMC5590826; doi:10.1371/journal.pone.0183901)
Supplement: S2 Fig — (A) Expression of miR-301a was determined in various tissues by quantitative real-time PCR indicates ubiquitous distribution of miR-301a, including significant expression in the heart (N = 3). Original uncropped blots are shown for Fig 4A (B), and 4D (C). (DOCX) [file pone.0183901.s002.docx]

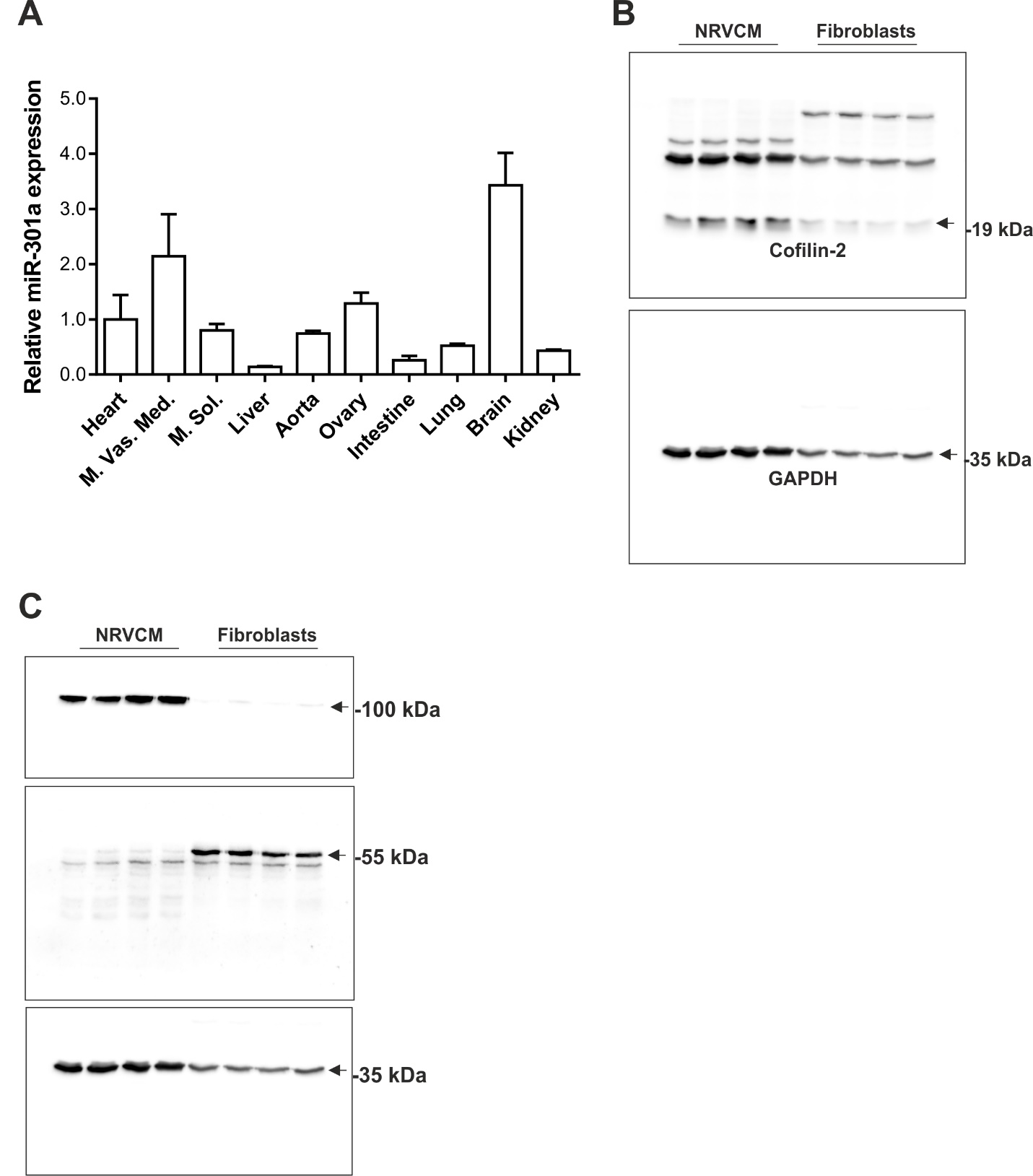


**Supplementary Fig. 2.** (**A**) Identification of miR-301a expression using quantitative real-time PCR across several tissues shows the ubiquitous distribution pattern. Original uncropped blots for Figure 4A (**B**), and 4D (**C**). L, protein ladder
