# Supplementary figures and images for: MicroRNA miR-301a is a novel cardiac regulator of Cofilin-2
Source: PLoS One. 2017 Sep 8;12(9):e0183901. doi: 10.1371/journal.pone.0183901 (PMC5590826; doi:10.1371/journal.pone.0183901)

**
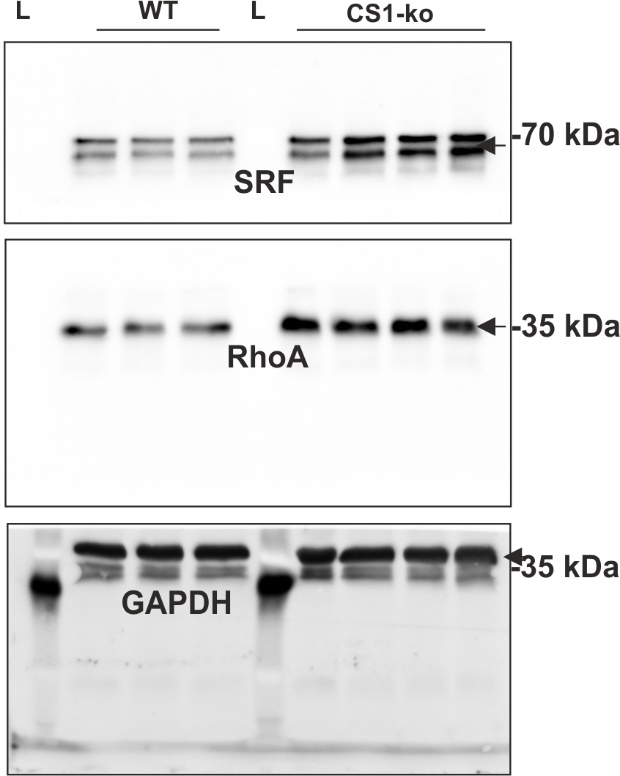
**

**Supplementary Fig. 3.** Original uncropped blots for Figure 6A. L, protein ladder

Supplement: S3 Fig — (DOCX) [file pone.0183901.s003.docx]
